# Supplementary material for: A live-cell biosensor of in vivo receptor tyrosine kinase activity reveals feedback regulation of a developmental gradient
Source: Cell Rep. Author manuscript; Available in PMC 2025 Aug 15. (PMC12356409; doi:10.1016/j.celrep.2025.115930)
Supplement: 1 [file NIHMS2099548-supplement-1.pdf]

Cell Reports, Volume *44*

Supplemental information

A live-cell biosensor of *in vivo* receptor tyrosine kinase activity reveals feedback regulation of a developmental gradient

Emily K. Ho, Rebecca P. Kim-Yip, Alison G. Simpkins, Payam E. Farahani, Harrison R. Oatman, Eszter Posfai, Stanislav Y. Shvartsman, and Jared E. Toettcher

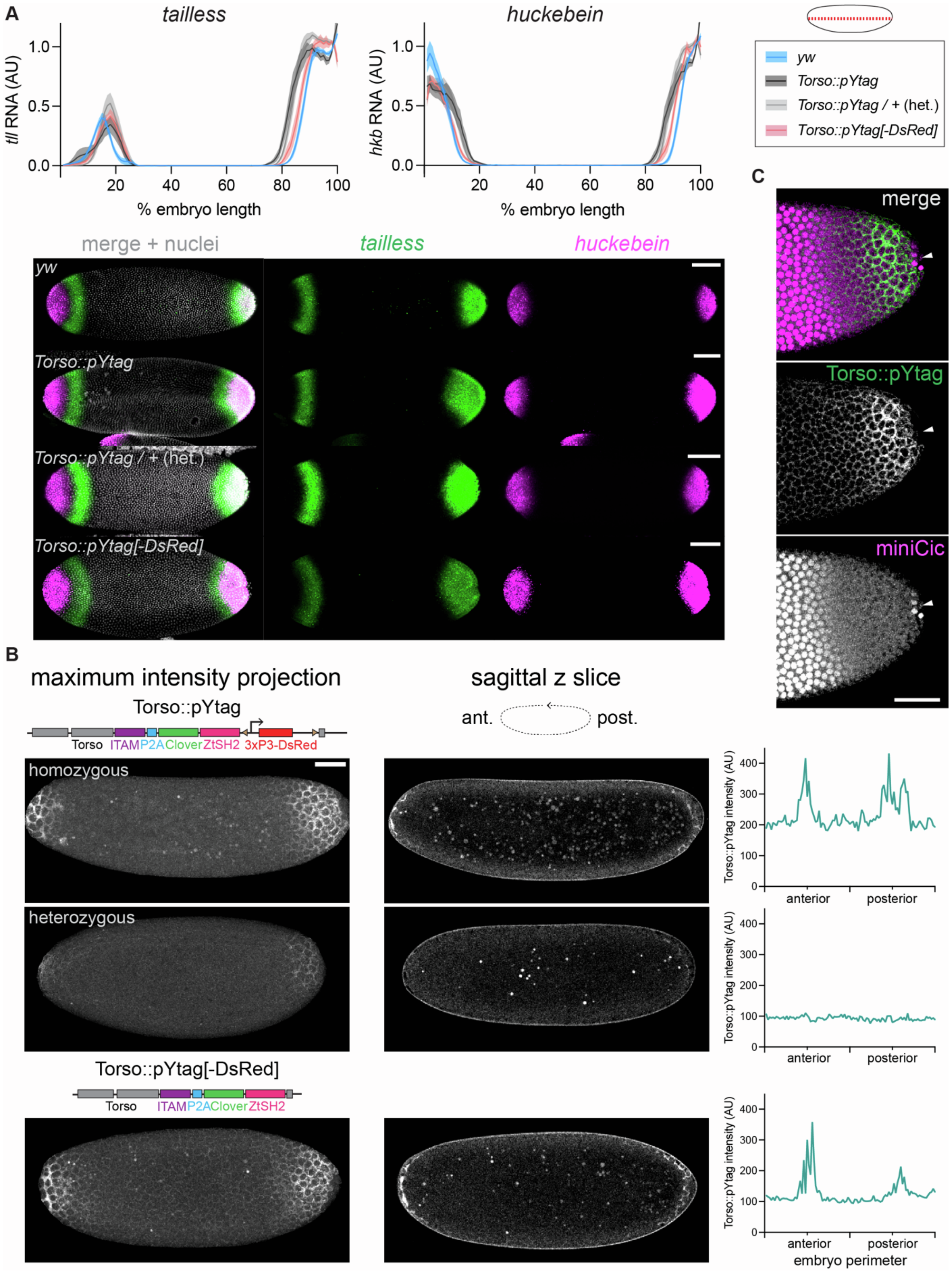
A
tailless
huckebein
tll RNA (AU)
hkb RNA (AU)
% embryo length
yw
Torso::pYtag
Torso::pYtag / + (het.)
Torso::pYtag[-DsRed]
merge + nuclei
tailless
huckebein
B
maximum intensity projection
sagittal z slice
Torso::pYtag
Torso
ITAM
P2A
Clover
ZtSH2
3xP3-DsRed
ant.
post.
homozygous
heterozygous
Torso::pYtag intensity (AU)
anterior
posterior
Torso::pYtag[-DsRed]
embryo perimeter
C
merge
Torso::pYtag
miniCic

Figure S1: Torso::pYtag detects Torso activity without significantly disrupting endogenous terminal patterning, related to Figure 1. (A) To assess terminal patterning in wild-type (*yw*), *Torso::pYtag*, *Torso::pYtag* / +, and *Torso::pYtag[-DsRed]* embryos, NC14 embryos were stained for *tll* and *hkb* mRNA using HCR FISH. Images show representative images and graphs show quantification across the anterior-posterior axis. The boundaries of *tll* and *hkb* expression, which are set by the ERK gradient, shift towards the center of the embryo in *Torso::pYtag* homozygous and heterozygous embryos but are largely restored to their wild-type positions when the 3xP3-DsRed marker is removed and only the pYtag is present. This result suggests that pYtag insertion influences terminal patterning, but this effect is largely due to the 3xP3-DsRed and not the pYtag itself. Lines are mean ± s.e.m. n = 20 (*yw*), 13 (*pYtag*), 12 (*pYtag/+*), 15 (*pYtag[-DsRed]*). Scale bar: 50 μm. (B) Comparison of Torso activity in *Torso::pYtag* and *Torso::pYtag[-DsRed]* embryos confirms that both genotypes had similar intensities and spatial patterns of Torso activity at the poles. Heterozygous embryos with only one copy of *Torso::pYtag* show a similar pattern but with reduced intensity. Images show NC12 embryos in two views. On the left are maximum intensity projections and on the right are sagittal z planes used for quantification. Bright foci in the center of the embryo are autofluorescent yolk granules, and the autofluorescent vitelline membrane is present in the sagittal images. Graphs show the Clover-ZtSH2 intensity around the perimeter for the pictured embryo of each genotype. Scale bar: 50 μm. (C) Repression of Torso activity in the pole cells is detectable by Torso::pYtag. Image shows the posterior pole of a NC13 embryo. Arrowhead denotes pole cells which have nuclear miniCic (ERK OFF) and no Clover-ZtSH2 signal. Scale bar: 50 μm.

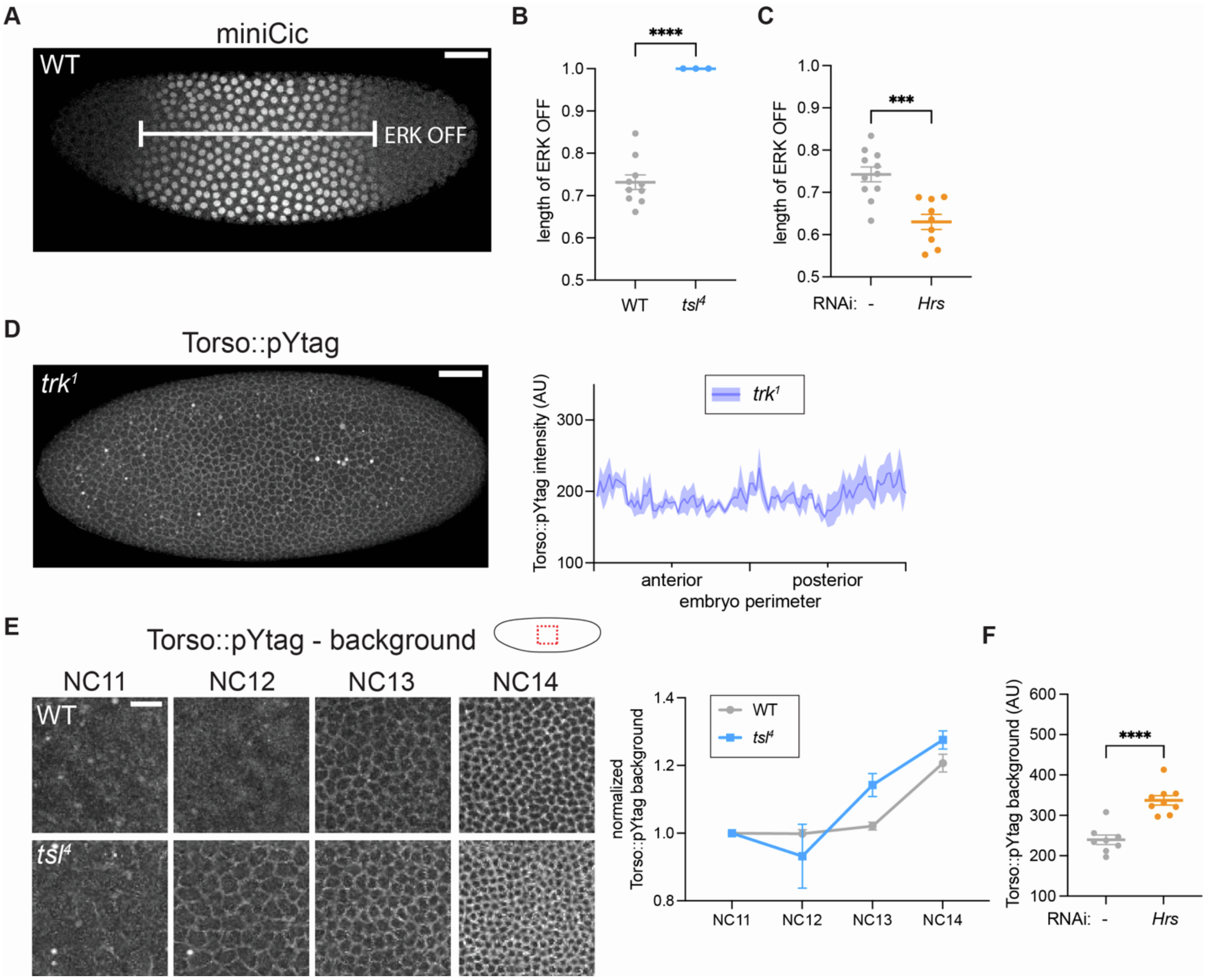

Figure S2: Validation of genetic perturbations to Torso activity, related to Figure 2. (A) To validate genetic perturbations to Torso, we sought a simple measure to compare ERK activity between embryos and conditions. The proportion of embryo length in which miniCic is nuclear reveals the "length of ERK OFF". The image shows an example of the "ERK OFF" portion of a wild-type NC12 embryo. Scale bar: 50 μm. (B) Comparison of ERK OFF embryo length in WT and *tsl*^{4} NC14 embryos. All *tsl*^{4} mutant embryos show nuclear miniCic throughout the entire embryo (see **Figure 2A**), indicating no active ERK signaling. Mean ± s.e.m., n = 10, 3 embryos. Unpaired t test. (C) Comparison of ERK OFF embryo length in control and *Hrs* RNAi NC14 embryos. The *Hrs* RNAi phenotype is variable, but there is a significant decrease in the ERK OFF length compared to controls, indicating higher ERK signaling. Mean ± s.e.m., n = 11, 9 embryos. Unpaired t test. (D) Torso::pYtag in a *trk*^{1} NC13 embryo shows no active Torso at the poles. Like in the *tsl*^{4} mutant, there is low level membrane localization of Torso::pYtag throughout the embryo. Scale bar: 50 μm. Graph shows quantification of Torso::pYtag around the perimeter of NC13 *trk*^{1} mutant embryos. Mean ± s.e.m. from n = 3 embryos. (E) Low-level localization of Clover-ZtSH2 to membranes in the center of the embryo where Torso is inactive, shown for WT and *tsl*^{4} embryos in NC11-14. Scale bar: 20 μm. Graph shows comparison of background Torso::pYtag intensity in WT and *tsl*^{4} embryos across NC11-14. Background intensities are normalized to the NC11 value. Mean ± s.e.m. from n = 9, 4 embryos. (F) Comparison of background Torso::pYtag intensity in control and *Hrs* RNAi NC13 embryos shows that *Hrs* RNAi embryos have significantly higher Torso::pYtag background. Mean ± s.e.m., n = 8,9 embryos. Unpaired t test. For all graphs, significance was defined as ***P<0.001 and ****P<0.0001.

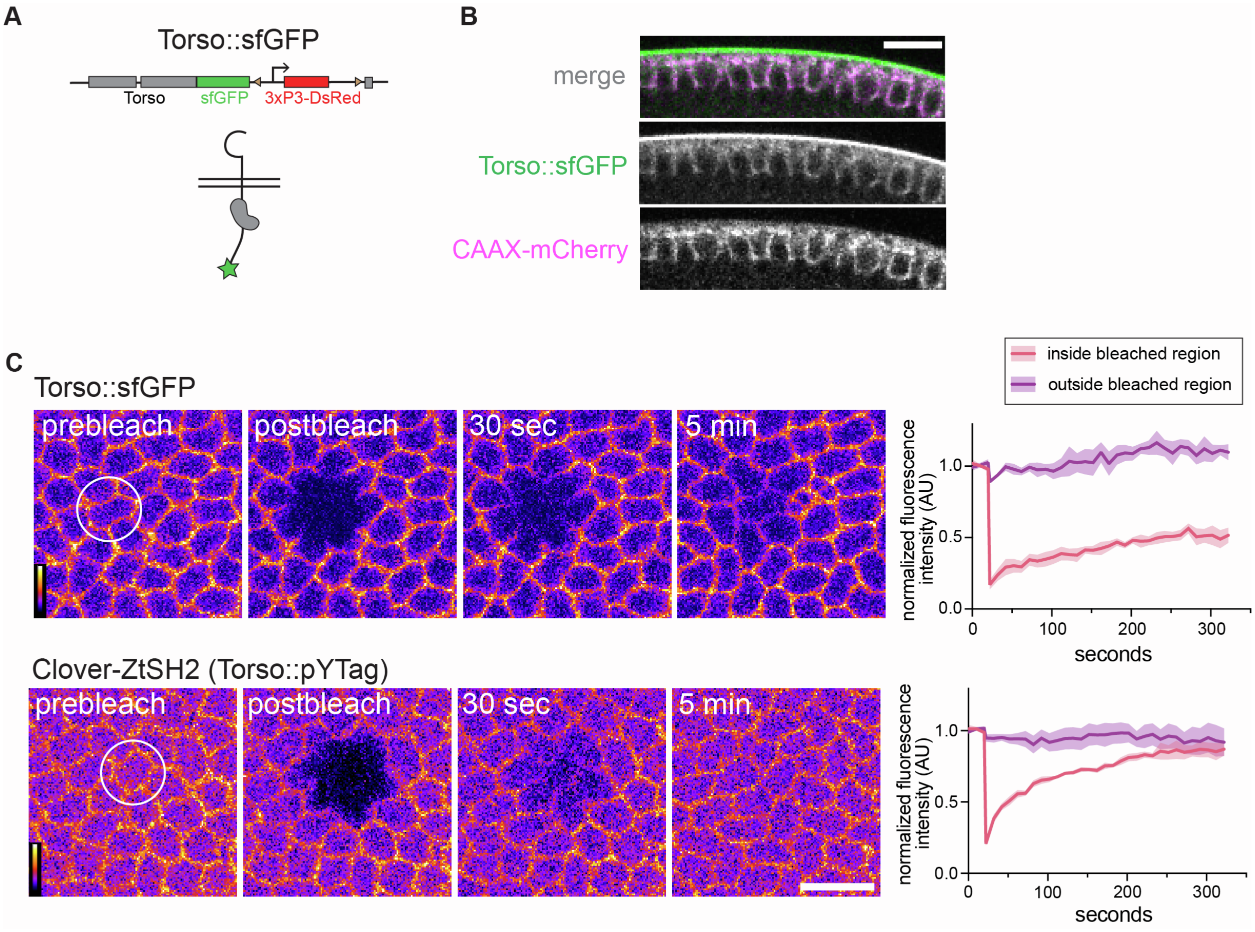

Figure S3: Torso localizes throughout the plasma membrane but its diffusion is restricted, related to Figure 2. (A) To visualize total Torso, we endogenously tagged the C-terminus of Torso with superfolder GFP (Torso::sfGFP). (B) Torso::sfGFP colocalizes with the plasma membrane marker CAAX-mCherry in a late NC14 embryo throughout both the apical and lateral domains of the membrane. Autofluorescence from the surrounding vitelline membrane is present in the Torso::sfGFP channel. Scale bar: 20 μm. (C) We used fluorescence recovery after photobleaching (FRAP) to assess diffusion of Torso (Torso::sfGFP) and Clover-ZtSH2 (Torso::pYtag) in NC13 embryos. Images show the region before and after photobleaching. The circle indicates the bleached region. Scale bar: 20 μm. Graphs show intensity within and outside the bleached region over the 5-minute recovery period. Mean ± s.e.m. n = 4 embryos.

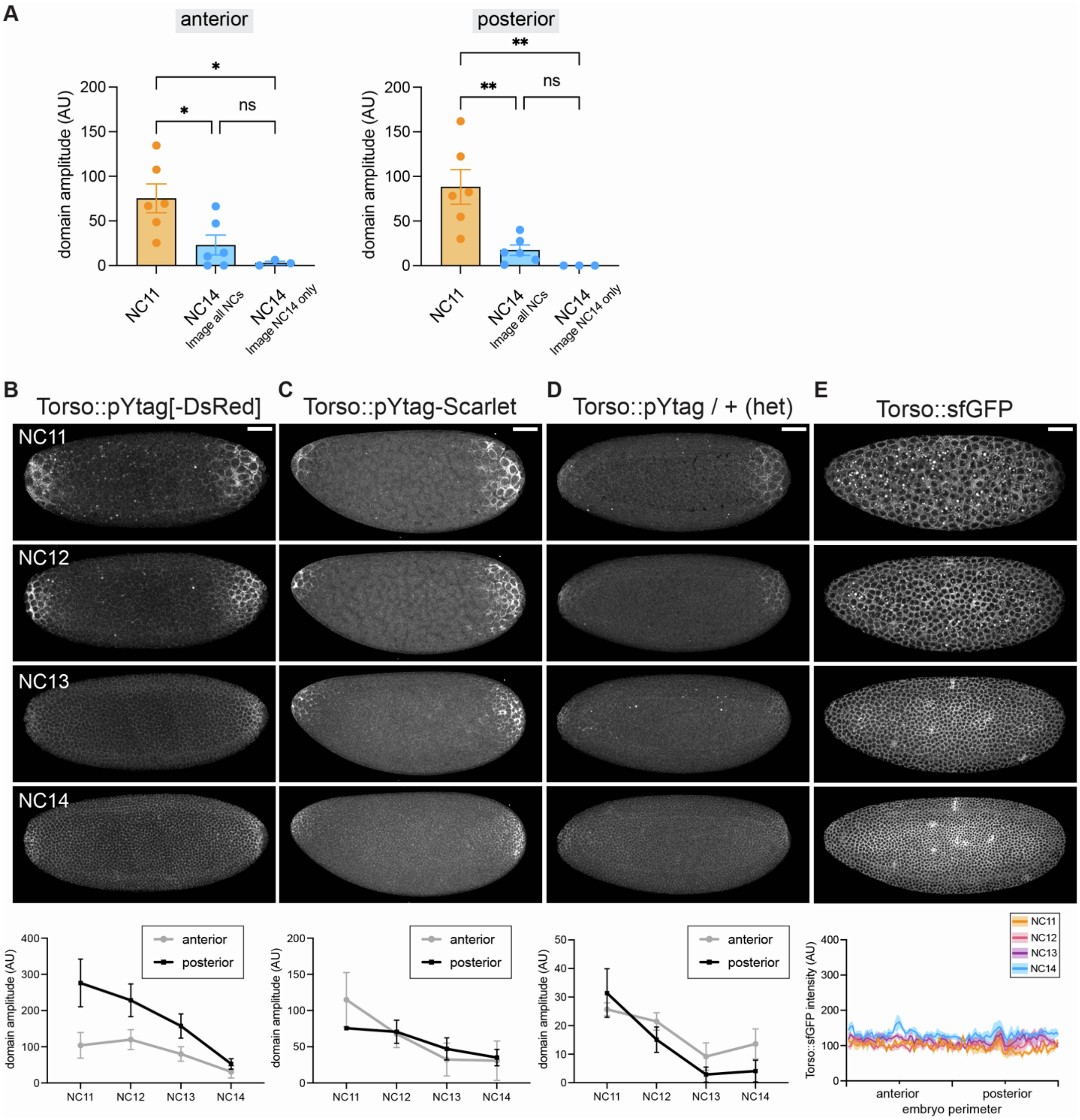

Figure S4: Validating the decrease in Torso activity over time, related to Figure 3. (A) To show that the decrease in Torso::pYtag intensity is not due to bleaching, NC14 embryos that were only imaged in NC14 were compared to embryos that had been imaged in NC11-14. There was no difference in these conditions, and both were significantly decreased compared to Torso::pYtag in NC11. RNAi control embryos were used for this experiment. Mean ± s.e.m. Significance from one-way ANOVA with Tukey's test. n = 3-6 embryos. Significance was defined as *P<0.05 and **P<0.01. n.s. indicates no significance. (B) Images show a *Torso::pYtag[-DsRed]* embryo over NC11-14. Quantification shows that Torso::pYtag domain amplitude decreases in the anterior and posterior. Mean ± s.e.m. n = 4. (C) Images show a *Torso::pYtag-Scarlet* embryo over NC11-14. Quantification shows that Torso::pYtag domain amplitude decreases in the anterior and posterior. Mean ± s.e.m. n = 7. (D) Images show a heterozygous *Torso::pYtag* embryo over NC11-14. Quantification shows that Torso::pYtag domain amplitude decreases in the anterior and posterior. Mean ± s.e.m. n = 4. (E) Images show a *Torso::sfGFP* embryo over NC11-14. Quantification of Torso::sfGFP

around the perimeter of a z slice shows that total Torso is slightly increasing over time. Mean ± s.e.m. n = 5. All scale bars: 50 μm.

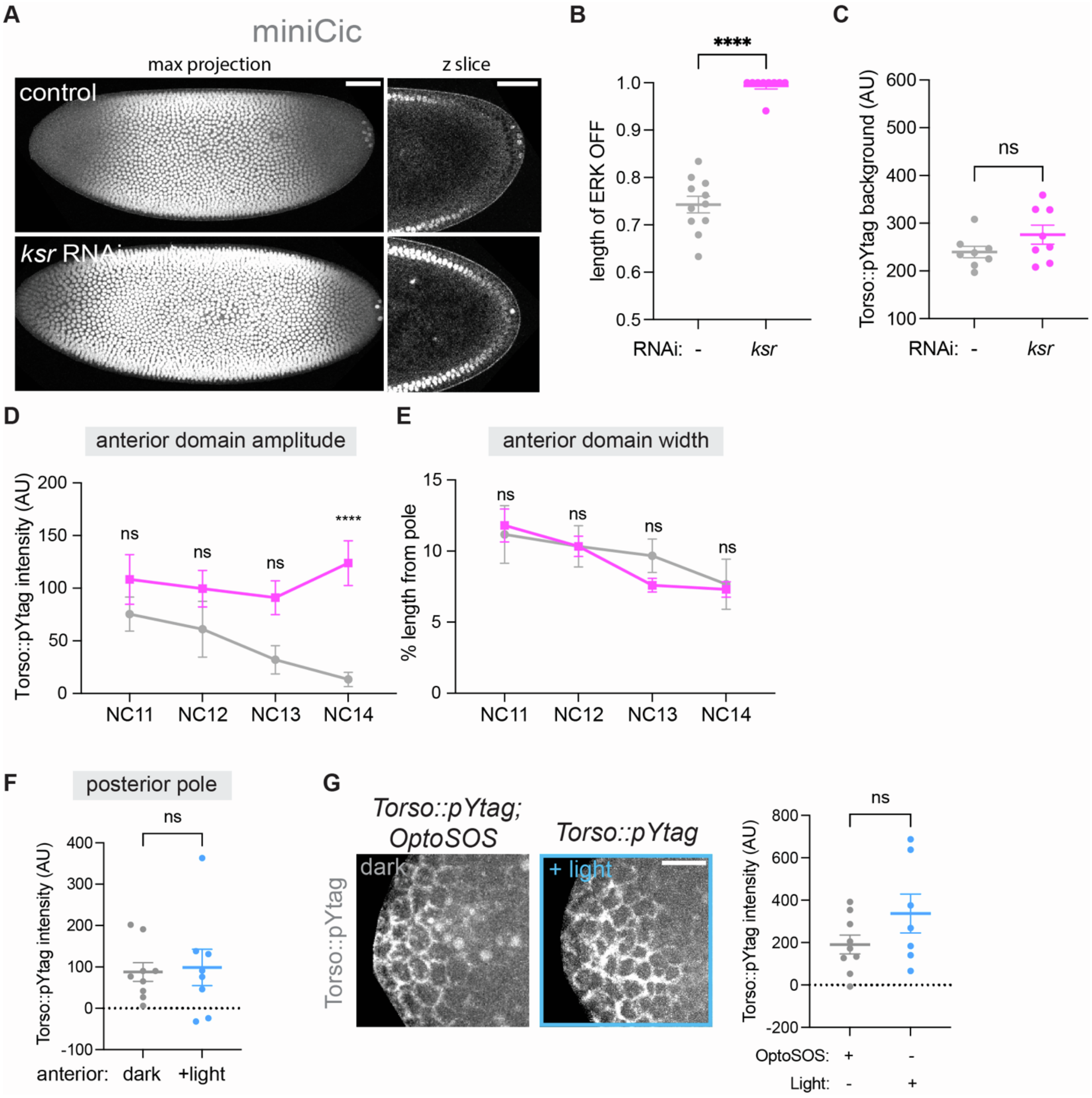

Figure S5: Validation of manipulations to assess negative feedback, related to Figure 4. (A) To validate that the *ksr* RNAi was effective in inhibiting ERK activity, we assessed miniCic activity in NC14 control and *ksr* RNAi embryos. While control embryos have cytoplasmic miniCic at the poles indicating that ERK is active, *ksr* RNAi embryos have nuclear miniCic throughout the embryo. We note that the nuclear intensity at the poles of *ksr* RNAi is lower than in the center of the embryo, suggesting there is a low level of residual ERK activity. However, the medial z slice shows that the signal is nuclear all the way to the pole, revealing that this residual ERK activity is very low. (B) Quantification of the ERK OFF embryo length in control and *ksr* RNAi NC14 embryos. Mean ± s.e.m. n = 11 (control), 9 (*ksr*) embryos. Significance by unpaired t test. (C) Graph shows no significant difference in the total Clover-ZtSH2 intensity of the inset region between genotypes, showing that background levels of Clover-ZtSH2 recruitment in NC13 do not change in *ksr* RNAi embryos. Mean ± s.e.m., n = 8 embryos in each condition. Unpaired t test. (D) Anterior domain amplitude over time for the same embryos as in (**Figure 4D**). The anterior pole shows a similar increase in Torso::pYtag activity in *ksr* RNAi embryos over time. Mean ± s.e.m. Significance by 2-way ANOVA with multiple comparisons test. (D) Anterior domain width over time for the same embryos as in

(**Figure 4E**). Mean ± s.e.m. Significance by 2-way ANOVA with multiple comparisons test. (E) Posterior Torso::pYtag intensity for the same embryos as in (**Figure 4H**). The posterior was unilluminated in all embryos and thus shows no difference between conditions. Mean ± s.e.m. n = 9. Unpaired t test. (F) To confirm that optogenetic stimulation with blue light did not bleach the Torso::pYtag, we illuminated Torso::pYtag embryos that did not express OptoSOS with the same illumination regime. There was no significant difference in anterior Torso::pYtag intensity between illuminated *Torso::pYtag* embryos and unilluminated *Torso::pYtag; OptoSOS* embryos. Mean ± s.e.m. n = 9 (unilluminated), 7 (illuminated). Unpaired t test. For all graphs, significance was defined as ****P<0.0001. ns indicates no significance.

Table S1. Primers used for CRISPR/Cas9 and genotyping, related to STAR Methods

Primer Name	Sequence
Torso 5' Homology F	CAAGAAGCGAATCTTTGAGAACAAGGAATACTTTGATTGCCTCGACTCATCGG
Torso 5' Homology R	ATTCAAAGGTTCTAGGTATAGCTCTTCCTCGCATGGCACTTGC
Torso 3' Homology F	TAGTCATTGCTTCAAGATTATAATGAACGAGTGCAATACATTCTAAATTCGAGTTCC
Torso 3' Homology R	CTAAGGGCGCTCAGGAGCTTTGGAATGGACACAACATCG
Torso Guide F	cttcgAGCAATGACTATTAATTCAA
Torso Guide R	aaacTTGAATTAATAGTCATTGCTc
Torso Genotyping F	AATAACCAATGCAGCCGACAACAAGGGCTATGGCCTGG
Torso Genotyping R	GGACTCTTTGGTTCCGTCACTTCGGAGTAGAGATCAGATATACTTCTCACC
EGFR 5' Homology F	TGAGTACAAGGCTGCTGGCGGCAAGATGCCCATCAAGTGG
EGFR 5' Homology R	CACCCTCGTCTCCGTGTTGCGGTTTCGATGCAGTGG
EGFR 3' Homology F	GCTCCAGTCGAGTAGGAGCAATTGCCAATGAAGAAGGAGAATCTTGCC
EGFR 3' Homology R	TTCTTGGCGGGCACCAACCGGTTATCAAGCC
EGFR PAM SDM F	AGACGAGaGTGGGCTCtGGtTCTCCACCTCCC
EGFR PAM SDM 5	AGCCCACtCTCGTCTCCGTGTTGCGGTTTCG
EGFR Guide F	cttcGAAACCGCAACACGGAGACG
EGFR Guide R	aaacCGTCTCCGTGTTGCGGTTTC
EGFR Genotyping F	AAGATCACCGACTTTGGGCTGGCCAAGTTGC
EGFR Genotyping R	ACGAAATACAGTTTGCGACCACGCCCTCTATAGAACAACC
Btl 5' Homology F	AAGATGGTCAAGGAGGAGCACACGGATACGGACATGG
Btl 5' Homology R	AGGTGTACTGATATCTCAGTGGAGACGTTTCCCGGAATGTTTCGGTGTCGGAGCC
Btl 3' Homology F	TTCGTAGTATAAGGAGACCAAAAAGAATTCCAACGAGTCAATCAGATCCCATCGAAGC
Btl 3' Homology R	GCTGTCAGGATCATCGTTAAGTTGGCTCCCCATTGTAATGAGTTCCTCG
Btl Guide F	cttcGAAACCGCAACACGGAGACG
Btl Guide R	aaacCGTCTCCGTGTTGCGGTTTC
Btl Genotyping F	AGCAGCTTAGCTTGGGCTCCATTTTGGGTGAGG
Btl Genotyping R	CGATAGATTCCCAACAAATTCAGGGGCATTTCCTCATCGAGCTG

Table S2. Fly genotypes used, related to STAR Methods

Figure	Maternal genotype
1D, 1E, S1A-C, 2A, 2B, 2E, 2F, 2H, S2A, S2B, S2E, S3C, 3B-E, S5G	Torso::pYtag, miniCic::mCherry
1D, 1E	EGFR::pYtag, miniCic::mCherry
1D, 1E	Zygotic genotype: btl-Gal4, UAS-CD4::mIFP / CyO ; btl::pYtag
S1A, 3B-E	yw
S1A, S1B, S4B	Torso::pYtag[-DsRed]; CAAX-mCherry
S1A, S1B, S4D	Torso::pYtag, miniCic::mCherry / +
2A, 2B, 2E, 2F, S2B, S2E	Torso::pYtag, miniCic::mCherry / Torso::pYtag ; tsl^4 / 15, tsl^4
2C, 2D, S2C, S2F, S4A, 4B-E, S5A-E	Torso::pYtag, 67 / Torso::pYtag, miniCic::mCherry ; 15 / +
2C, 2D, S2C, S2F	Torso::pYtag, 67 / Torso::pYtag, miniCic::mCherry ; 15 / UAS-Hrs RNAi
2G	Torso::pYtag[-ITAM], miniCic::mCherry
2H, S3B	Torso::sfGFP / + ; CAAX-mCherry / +
S2D	trk^1, Torso::pYtag
S3C, S4E	Torso::sfGFP / CyO
3B-E	miniCic::mCherry / + ; His2Av-GFP / +
S4C	Torso::pYtag-Scarlet, miniCic::mNeonGreen
4B-E, S5A-E	Torso::pYtag, 67 / Torso::pYtag, miniCic::mCherry ; 15 / UAS-ksr RNAi
4G, 4H, S5F, S5G	Torso::pYtag, 67 / Torso::pYtag ; 15 / UAS-optoSOS
5A, 5B	Torso::pYtag, MCP::mCherry / Torso::pYtag x Sp/CyO ; tll-MS2 males
